# Glutamate Imaging Reveals Multiple Sites of Stochastic Release in the CA3 Giant Mossy Fiber Boutons

**DOI:** 10.3389/fncel.2019.00243

**Published:** 2019-06-04

**Authors:** Sylvain Rama, Thomas P. Jensen, Dmitri A. Rusakov

**Affiliations:** UCL Queen Square Institute of Neurology, University College London, London, United Kingdom

**Keywords:** dentate gyrus, CA3 pyramidal cell, short-term plasticity, glutamate release, giant mossy fiber bouton, action potential

## Abstract

One of the most studied central synapses which have provided fundamental insights into cellular mechanisms of neural connectivity is the “giant” excitatory connection between hippocampal mossy fibers (MFs) and CA3 pyramidal cells. Its large presynaptic bouton features multiple release sites and is densely packed with thousands of synaptic vesicles, to sustain a highly facilitating “detonator” transmission. However, whether glutamate release sites at this synapse act independently, in a stochastic manner, or rather synchronously, remains poorly understood. This knowledge is critical for a better understanding of mechanisms underpinning presynaptic plasticity and postsynaptic signal integration rules. Here, we use the optical glutamate sensor SF-iGluSnFR and the intracellular Ca^2+^ indicator Cal-590 to monitor spike-evoked glutamate release and presynaptic calcium entry in MF boutons. Multiplexed imaging reveals that distinct sites in individual MF giant boutons release glutamate in a probabilistic fashion, also showing use-dependent short-term facilitation. The present approach provides novel insights into the basic mechanisms of neurotransmitter release at excitatory synapses.

## Introduction

The dentate gyrus is the entry into the hippocampus, with the mossy fibers (axons of granule cells) innervating both CA3 pyramidal cells and stratum-lacunosum interneurons (Acsády et al., 1998). These distinct postsynaptic cell populations are connected through distinct presynaptic elements: “giant” mossy fiber boutons (gMFBs, 4–10 μm) across and their smaller (2–3 μm) variant synapsing onto the thorny excrescences of CA3 pyramidal cells, and relatively small (0.5–2 μm) en-passant boutons and the filopodial extensions emerging from gMFBs both connecting to interneurons (Chicurel and Harris, 1992; Acsády et al., 1998; Rollenhagen et al., 2007). Hippocampal MFB connections show prominent facilitation during repetitive activity and are considered strong “detonating” synapses, generating large postsynaptic responses in CA3 pyramidal cells (Vyleta et al., 2016). This function is sustained by specific morphology, as they show multiple active zones per MFB, and thousands of synaptic vesicles densely packed inside (Acsády et al., 1998; Rollenhagen et al., 2007). They have been widely studied because MF synapses play a key role in processing spatial information such as pattern completion, pattern separation and storage of sequences of events (Kobayashi and Poo, 2004; Bischofberger et al., 2006). Moreover, these synapses provide a key experimental model for analog-digital control of synaptic transmission, linking presynaptic voltage, presynaptic calcium entry, and glutamate release (Geiger and Jonas, 2000; Alle and Geiger, 2008; Scott et al., 2008). Finally, changes in MF transmission have been a major pathophysiological indicator in epilepsy (Ben-Ari and Represa, 1990; Morimoto et al., 2004) and Down syndrome (Witton et al., 2015). Therefore, an inquisitive exploration of MF synapses helps to understand the fundamentals of synaptic transmission and hippocampal network function in a wide context.

## Results

### Simultaneous Imaging of Presynaptic Calcium Entry and Glutamate Release at Presynaptic MF Boutons

**FIGURE 1:**
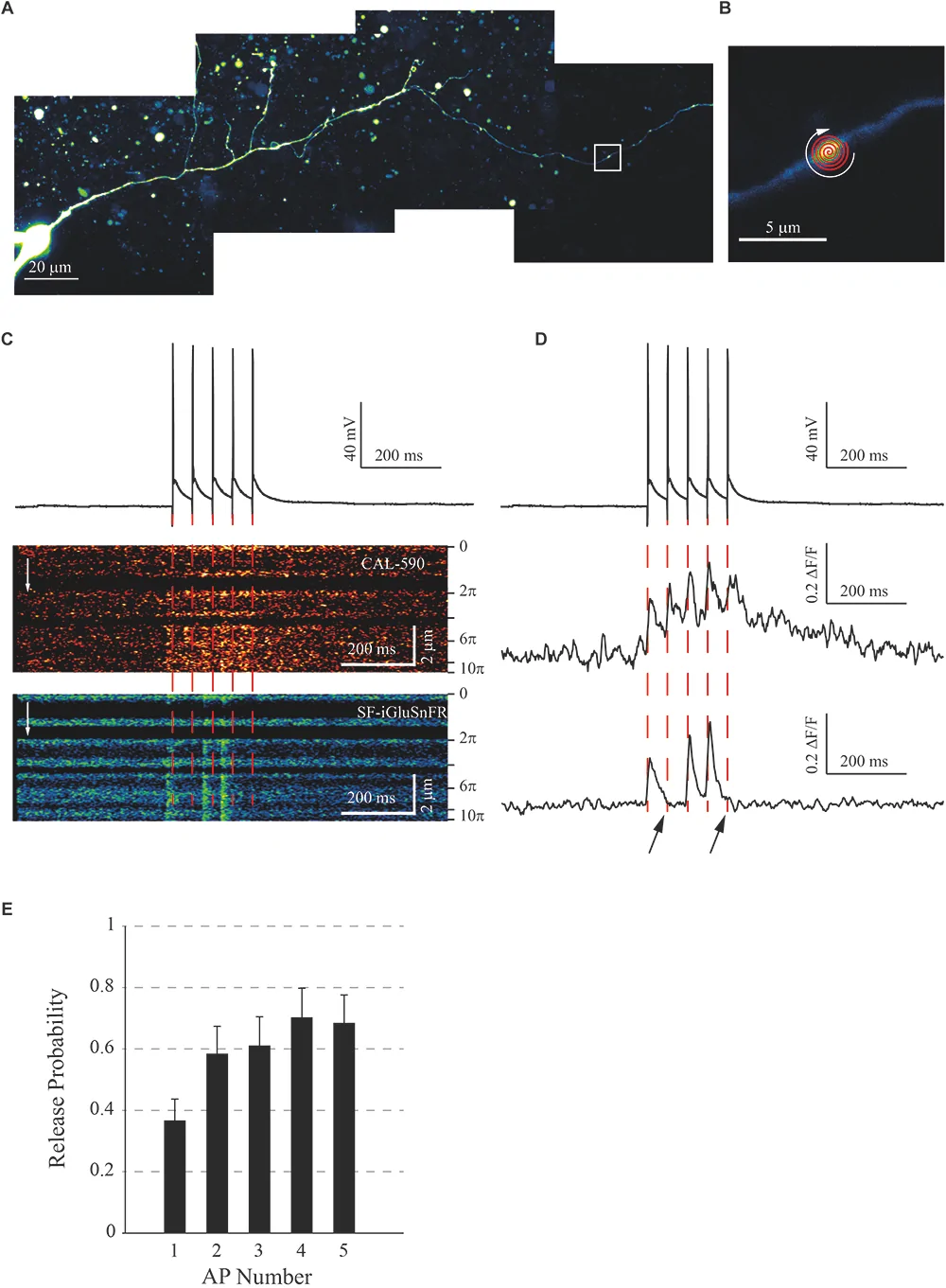
Experimental Protocol. **(A)** Dentate gyrus granule cell expressing SF-iGluSnFR A184V, maintained in current-clamp. Collage of 20 μm deep image stacks, axon was followed to the bouton of interest (white square). **(B)** Area shown by the white square in panel **(A)**. A small varicosity (<2μm) reveals a typical en-passant bouton. The fast “Tornado” (spiral) line-scan was set-up to cover most of the bouton visible area. **(C)** Typical imaging protocol. Traces: Five APs initiated by brief (5 ms) current pulses at 20 Hz (holding voltage −80 mV); Image panels: Tornado line-scans recorded in the Cal-590 (upper) and SF-iGluSnFR (lower) emission channels. **(D)** Same recordings as in panel **(C)**, but pixel values were averaged over the length of the tornado line-scan and converted as ΔF/F. Note that for the Cal-590 signal, each AP induced a calcium entry in the presynaptic bouton (middle) whereas APs 2 and 5 failed to induce a glutamate release (bottom, black arrows). **(E)** Average release probability (mean ± SEM) for the AP train (*n* = 11 boutons). Note the increasing probability with the AP number, reflecting presynaptic facilitation.

To directly monitor glutamate release by MFBs, we turned to organotypic slices which we biolistically transfected with the SF-iGluSnFR A184V construct (termed iGluSnFR thereafter; see section “Methods”), as detailed earlier (Jensen et al., 2019). With the sparse expression among cells, the iGluSnFR basal fluorescence was sufficient to reveal cell morphology and to track the cell axon. We thus patch-loaded dentate granule cells with the red-shifted calcium-sensitive dye Cal-590 (Tischbirek et al., 2015; Jensen et al., 2019) and followed their axon up to at least 100 μm from the cell soma, toward the CA3 area (Figures 1A,B).

In individual cells, we evoked 5 action potentials (APs) at 20 Hz, a bursting pattern similar to that *in vivo* (Pernía-Andrade and Jonas, 2014; Vyleta et al., 2016; Chamberland et al., 2018). Once we focused on the MF bouton of interest, either of the two imaging protocols was employed: (i) for boutons smaller than 4 μm, a fast “tornado” scanning mode covering the bouton profile (Jensen et al., 2017, 2019), and (ii) for boutons above 4 μm, a straight line-scan along the longest axis of the bouton. In either case, we scanned at 500 Hz and collected both iGluSnFR and Cal-590 fluorescence. Signals were converted as *ΔF/F* values, and the Cal-590 signal was used to confirm AP arrival to the bouton (Figure 1C). The iGluSnFR signal revealed glutamate release sites in the bouton, showing release successes and failures (Figure 1D). At each individual site, release probability (calculated as the release success rate over all the trials) increased progressively with the AP number in the train, showing classical facilitation (Figure 1E). The initial release probability (first AP) at individual release sites was 0.37 ± 0.07 (mean ± SEM, *n* = 11 boutons), consistent with previous observations (Lawrence et al., 2004).

### Distinct Release Sites Display Stochastic Release

**FIGURE 2:**
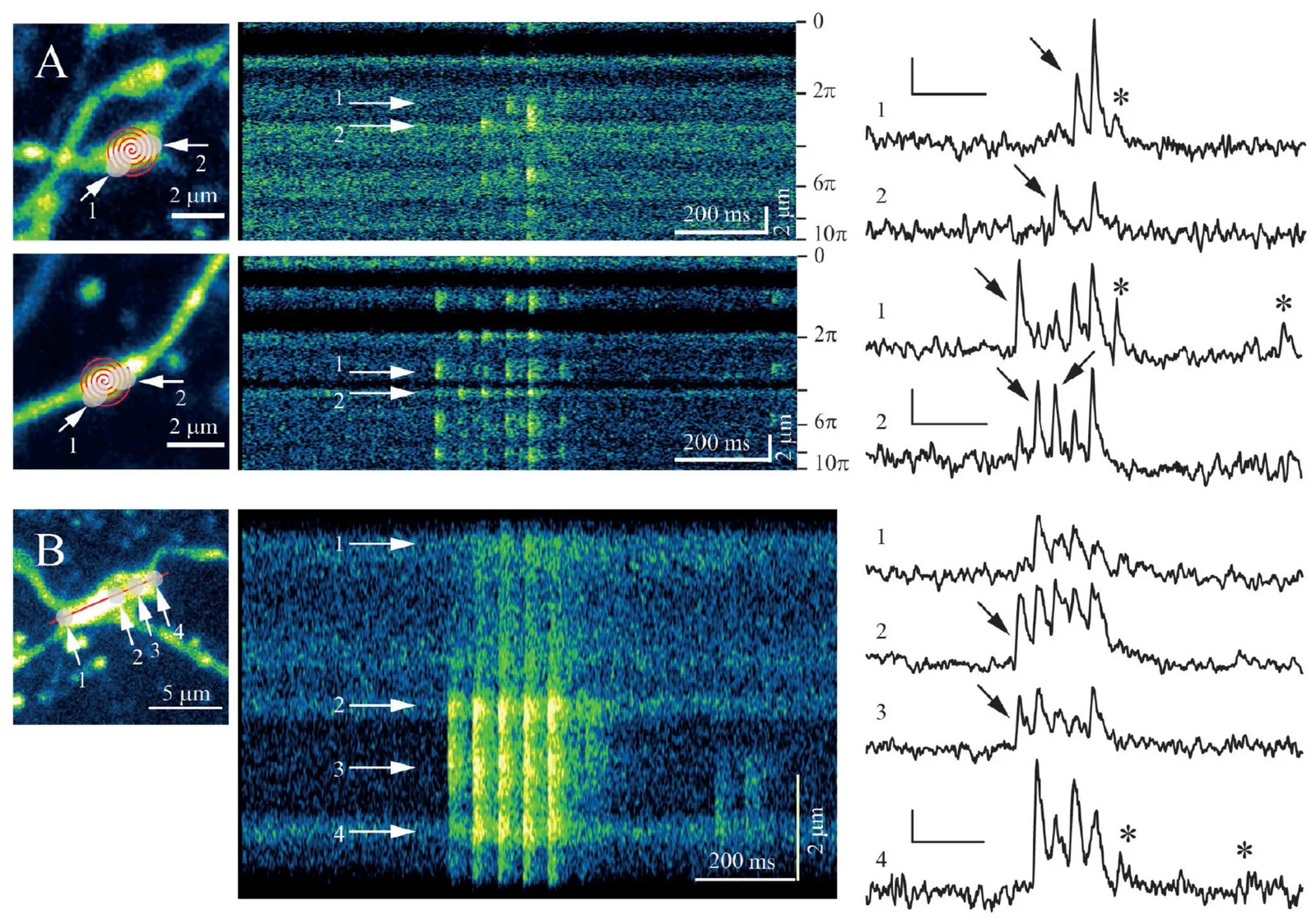
Monitoring distinct glutamate release sites in MFBs. **(A)** Two examples of large (>2μm) MF boutons showing multiple un-synchronized release sites. Left panels: Z-projection of a 3D stack, with Tornado line scan superimposition (red); arrows 1 and 2, locations of glutamate-releasing sites (white transparent areas). Middle panels: tornado line-scan of the iGluSnFR signal, showing un-synchronized releases of glutamate. Note the repeating patterns, as the tornado line-scan enters and exits the area periodically, at each turn of the spiral. Right: ΔF/F traces for areas 1 and 2, as indicated. Note unsynchronized peaks of glutamate release during APs (black arrows), and some spontaneous releases (asterisks). Scale Bars: 0.2 ΔF/F and 200 ms. **(B)** Example of line-scan acquisition on a giant (>5μm) MB bouton. Left: Z-projection of a 3D stack, with line-scan superimposition (red). Other notations as in panel **(A)**. Scale Bars: 0.2 ΔF/F and 200 ms.

In 6 out of 11 recorded boutons, we could clearly distinguish at least two active release sites, providing rapid glutamate discharges, which did not appear synchronized. We recorded between 3 and 14 consecutive trials (1 min apart) per bouton. Glutamate releases from spatially separate areas were detected in 80 ± 12% trials per bouton (mean ± SEM, a total of *n* = 43 trials). They were either synchronized or independent (Figure 2A). In 25 ± 8% of the recordings (*n* = 43), we could observe spontaneous (or possibly asynchronous, post-burst) release by some release sites (asterisks in Figure 2 traces). In one gMFB, there were up to four distinct release sites with the first AP inducing glutamate release in only 2 of them and spontaneous release in one of them, independent from the other 3 (Figure 2B).

## Discussion

In this brief report, we expressed the SF-iGluSnFR reporter dye in dentate granule cells and monitored glutamate release in gMFBs during brief trains of evoked APs. We detected multiple release sites in individual boutons displaying non-synchronized release activity. This appears in contrast to CA3-CA1 synapses, the majority of which displayed only one detectable glutamate release hotspot (20 out of 23; organotypic slices) whereas the two-hotspot boutons (3 out of 23) showed no detectable asynchrony (Jensen et al., 2019). At the same time, EM data report dual active zones in 38% of CA3-CA1 connections (Shepherd and Harris, 1998), suggesting that super-resolution approaches, such as stochastic localization, once combined with the present technique, might help to better resolve individual release hotspots. Indeed, optical diffraction limit, stereological bias, and limitation of 3D volume scanning should underestimate the occurrence of release sites and therefore their release asynchrony. In some cases, the distinction between en-passant boutons [which are not supposed to have multiple active zones (Acsády et al., 1998)] and the small subtype of giant MF boutons could also be less than clear-cut. Despite of these limitations, the present approach enables us to detect and explore glutamate release and its stochastic features in a direct manner, the task that has hitherto been difficult to achieve.

### Maintenance of High Frequency Transmission by Cross-Talk?

At many synapses, the rates of vesicle fusion exceed the rates of their replenishment leading to vesicle depletion during high-frequency activity (Zucker and Regehr, 2002). However, gMFBs could have formed in a similar way to the Purkinje terminals or the neuro-muscular junction (Telgkamp et al., 2004; Knodel et al., 2014), with large boutons with multiple active zones and densely packed with synaptic vesicles (Rollenhagen and Lübke, 2010). At gMFB synapses, large areas of the opposing pre- and postsynaptic membranes are separated by a narrow cleft. This extracellular space geometry provides, at least in theory, favorable conditions for neurotransmitter spill-over among multiple postsynaptic densities, thus achieving high-fidelity transmission even at low release probabilities and low rates of vesicle replenishment (Vergnano et al., 2014; Zheng et al., 2017). However, our glutamate imaging data suggest that this may not be the case for the MF-CA3 transmission, as the majority of individual glutamate release hotspots in presynaptic boutons do not seem to overlap, at least during first spikes. Indeed, if Purkinje cells do fire at high rates (Telgkamp et al., 2004), it is rarely the case for granule cells of the dentate gyrus (Pernía-Andrade and Jonas, 2014). Moreover, lower release probabilities and readily releasable vesicle pools make hippocampal mossy fiber boutons more suitable for presynaptic facilitation than high-frequency transmission (Delvendahl et al., 2013).

### Implications for Postsynaptic Signal Integration and Presynaptic Machinery

Morphological studies employing 3D electron microscopy have shown that individual giant MF boutons tend to form synaptic connections on more than one postsynaptic CA3 pyramidal cell (Acsády et al., 1998; Rollenhagen and Lübke, 2010). The present finding that individual release sites in such boutons can discharge glutamate relatively independently in a stochastic fashion, suggests that postsynaptic responses in their distinct cell targets could be similarly de-synchronized. It has been previously reported that in juvenile rats, gMFBs may co-release GABA and glutamate (Walker et al., 2001; Ruiz et al., 2003; Beltrán and Gutiérrez, 2012; Münster-Wandowski et al., 2013). The present results thus suggest that GABA and glutamate release from the same gMFB could be, in principle, stochastically separated. Finally, gMFBs show significant structural plasticity in the pilocarpine and kindling models of epilepsy (Danzer et al., 2010; McAuliffe et al., 2011), which could directly affect functional interaction between their individual release sites. What molecular mechanisms underpin such structural and functional changes remains an intriguing and important question.

## Methods

### Organotypic Cultures of Rat Hippocampus

Hippocampal slice cultures were prepared as described previously (Bialowas et al., 2014). All experiments were carried out according in accordance with the European Commission Directive (86/609/EEC) and the United Kingdom Home Office (Scientific Procedures) Act (1986). Briefly, postnatal day 7–8 Wistar rats were briefly anesthetized by isoflurane inhalation, the brain removed and each hippocampus individually dissected in ice-cold sterile slicing solution consisting (in mM) of Sucrose 105, NaCl 50, KCl 2.5, NaH2PO4 1.25, MgCl2 7, CaCl2 0.5, Ascorbic acid 1.3, Sodium pyruvate 3, NaHCO3 26 and Glucose 10. Hippocampal slices (350 μm) were placed on 20-mm latex membranes (Millicell-CM, Millipore, United Kingdom) inserted into 35-mm Petri dishes containing 1 ml of culture medium and maintained for up to 30 days in an incubator at 34°C, 95% O2–5% CO_2_. The culture medium contained (in ml) 25 minimal essential medium, 12.5 Hanks’ balanced saline solution, 12.5 horse serum, 0.5 penicillin/streptomycin, 0.8 glucose (1 M), 0.1 ascorbic acid (1 mg/ml), 0.4 HEPES (1 M), 0.5 B27, and 8.95 sterile H_2_O. To limit glial proliferation, 5 mM cytosine-arabinoside (Ara-C) was added to the culture medium at 4 days *in vitro* (DIV) for one night.

### Biolistic Transfection of iGluSnFR Variants

Second generation iGluSnFR variant SF-iGluSnFR.A184V was expressed under a synapsin promoter in dentate gyrus granule cells in organotypic slice cultures using biolistic transfection techniques adapted from manufacturer’s instructions. In brief, 6.25 mg of 1.6 micron Gold micro-carriers were coated with 30 μg of SF-iGluSnFR plasmid. Organotypic slice cultures at 8 DIV were shot using the Helios gene-gun system (Bio-Rad) at 120 psi. The slices were then returned to standard culture media the next day and remained for 3–7 days before experiments were carried out.

### Axon Tracing and Imaging of Pre-synaptic Boutons

We used a Femtonics Femto2D-FLIM imaging system, integrated with patch-clamp electrophysiology (Femtonics, Budapest) and linked on the same light path to two femtosecond pulse lasers MaiTai (SpectraPhysics-Newport) with independent shutter and intensity control. Patch pipettes were prepared with thin walled borosilicate glass capillaries (GC150-TF, Harvard apparatus) with open tip resistances 2.5–3.5 MΩ. Internal solution contained (in mM) 135 potassium methanesulfonate, 10 HEPES, 10 di-Tris-Phosphocreatine, 4 MgCl2, 4 Na2-ATP, 0.4 Na-GTP (pH adjusted to 7.2 using KOH, osmolarity 290–295), and supplemented with Cal-590 (300 μM; AAT Bioquest).

Pre-synaptic imaging was carried out using an adaptation of pre-synaptic glutamate and Ca^2+^ imaging methods previously described (Jensen et al., 2017, 2019). Cells were first identified as iGluSnFR expressing using two-photon imaging at 910 nm and patched in whole cell mode as above. Following break-in, 10–15 min were allowed for Cal-590 to equilibrate across the axonal arbor. Axons, identified by their smooth morphology and often tortuous trajectory, were followed in frame scan mode to their targets. Putative Giant Boutons were identified as varicosities on axon collaterals with a minimum diameter and length of ∼2–3 μm (Acsády et al., 1998; Rusakov and Fine, 2003; Rama et al., 2015).

For fast imaging of action-potential mediated iGluSnFR and Cal-590 fluorescence transients at single boutons a spiral shaped (“tornado”) scan line was placed over the bouton of interest (described further in the text), which was then scanned at a sampling frequency of ∼500 Hz with excitation at 910 nm. APs were typically induced by trains (20 Hz) of 5 short (5 ms) pulses of depolarizing current (900–1400 pA) in current clamp mode and synchronized with biphoton fluorescent imaging. Ten to fifteen acquisitions were made per boutons, recordings showing APs failures were discarded.

### Data Analysis

After acquisition, iGluSnFr or Cal-590 fluorescence was converted to *ΔF/F* values. Region of Interest (ROIs) were defined by fitting the fluorescence profile with Gaussian equation and extracting the Full Width at Half Maximum (FWHM) of this Gaussian. All of this was calculated by custom-written analysis programs written in Labview (National Instruments) and Matlab (MathWorks).

## Data Availability

All datasets generated for this study are included in the manuscript and/or the supplementary files.

## Ethics Statement

All experiments were carried out according in accordance with the European Commission Directive (86/609/EEC) and the United Kingdom Home Office (Scientific Procedures) Act (1986).

## Author Contributions

SR, TJ, and DR designed the experiments. SR and TJ carried out the patch-clamp and imaging experiments in neurons. SR carried out the analysis. SR and DR wrote the manuscript. All authors contributed to manuscript writing.

## Conflict of Interest Statement

The authors declare that the research was conducted in the absence of any commercial or financial relationships that could be construed as a potential conflict of interest.
