# Correction: Glutamate imaging reveals multiple sites of stochastic release in the CA3 giant mossy fiber boutons

**DOI:** 10.3389/fncel.2026.1932278

**Published:** 2026-07-30

**Authors:** Sylvain Rama, Thomas P. Jensen, Dmitri A. Rusakov

**Affiliations:** UCL Queen Square Institute of Neurology, University College London, London, United Kingdom

**Keywords:** dentate gyrus, CA3 pyramidal cell, short-term plasticity, glutamate release, giant mossy fiber bouton, action potential

There was a mistake in Figure 2: it appears heavily distorted in the ONLINE version of the article. The corrected figure appears below (and in the PDF version of the article).

**Figure 2:**
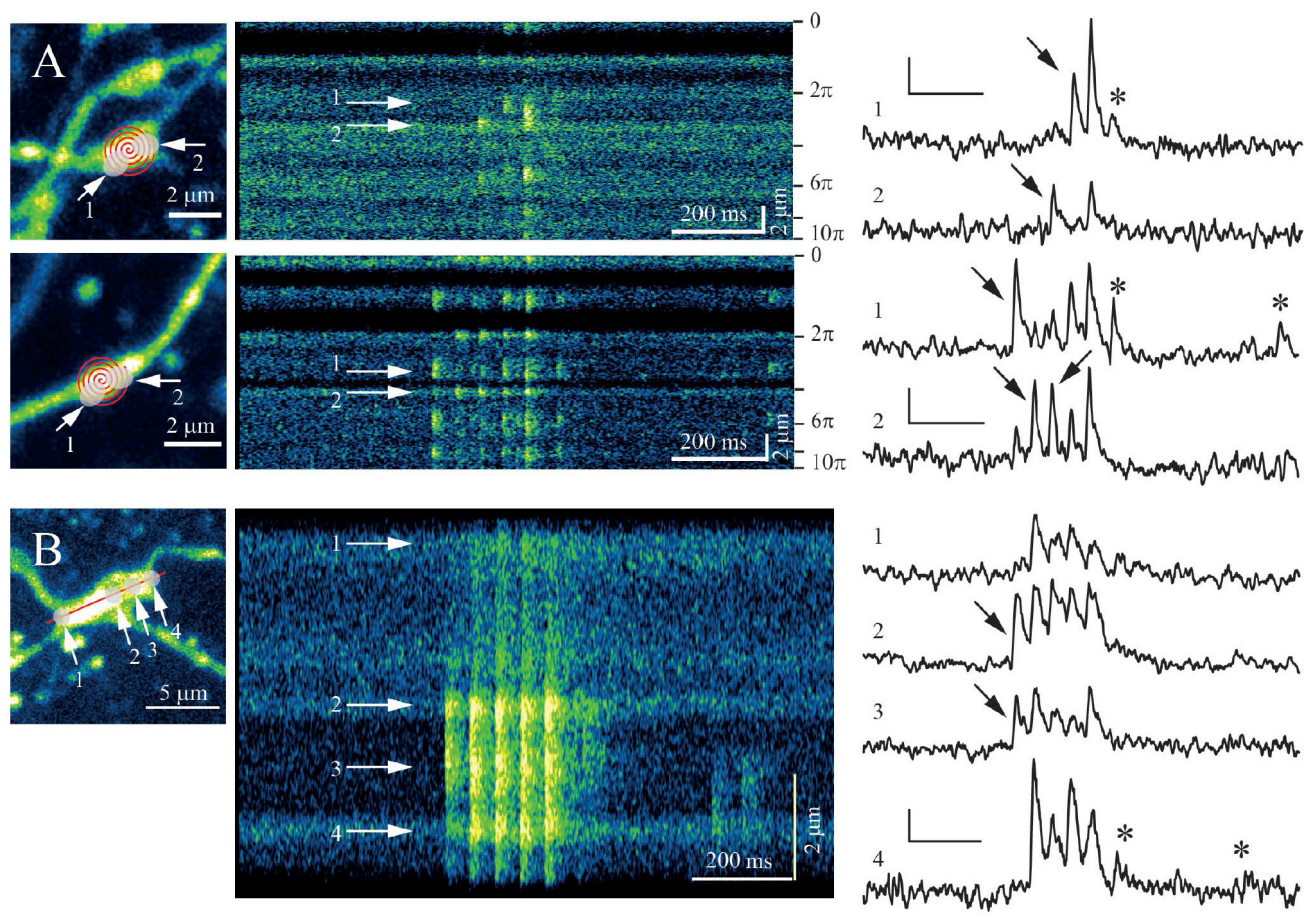
Monitoring distinct glutamate release sites in MFBs. **(A)** Two examples of large (>2 μm) MF boutons showing multiple un-synchronized release sites. Left panels: Z-projection of a 3D stack, with Tornado line scan superimposition (red); arrows 1 and 2, locations of glutamate-releasing sites (white transparent areas). Middle panels: tornado line-scan of the iGluSnFR signal, showing un-synchronized releases of glutamate. Note the repeating patterns, as the tornado line-scan enters and exits the area periodically, at each turn of the spiral. Right: ΔF/F traces for areas 1 and 2, as indicated. Note unsynchronized peaks of glutamate release during APs (black arrows), and some spontaneous releases (asterisks). Scale Bars: 0.2 ΔF/F and 200 ms. **(B)** Example of line-scan acquisition on a giant (>5 μm) MB bouton. Left: Z-projection of a 3D stack, with line-scan superimposition (red). Other notations as in panel **(A)**. Scale Bars: 0.2 ΔF/F and 200 ms.

The original version of this article has been updated.

